# Octopamine modulates the innate immune response in *Drosophila melanogaster*

**DOI:** 10.3389/fimmu.2025.1720126

**Published:** 2026-01-08

**Authors:** Karin Uliczka, Stephanie Papenmeier, Beate Höschler, Christina Wagner, Thomas Roeder, Holger Heine

**Affiliations:** 1 Division of Innate Immunity, Priority Research Area Chronic Lung Diseases, Research Center Borstel - Leibniz Lung Center, Borstel, Germany; 2 Airway Research Center North (ARCN), Member of the German Center for Lung Research (DZL), Borstel, Germany; 3 Division of Invertebrate Models, Priority Research Area Chronic Lung Diseases, Research Center Borstel - Leibniz Lung Center, Borstel, Germany; 4 Department of Molecular Physiology, Zoology, Kiel University, Kiel, Germany

**Keywords:** antimicrobial peptide, biogenic amine, *Drosophila* immunity, hemocyte, octopamine, phagocytosis

## Abstract

**Background:**

Octopamine, the functional equivalent of noradrenaline in invertebrates, is a key neuromodulator that also influences immune functions. However, the receptor‑specific roles of octopamine in regulating innate immunity in *Drosophila melanogaster* remain incompletely understood.

**Methods:**

Using loss‑of‑function mutants for the two major octopamine receptors Octβ1R and Octβ2R, combined with systemic bacterial infection models, bacterial clearance assays, in vivo and in vitro phagocytosis assays, and quantification of antimicrobial peptide (AMP) gene expression, we dissected the contribution of each receptor to distinct immune effector modules.

**Results:**

Flies deficient in Octβ1R or Octβ2R exhibited reduced survival and impaired bacterial clearance following systemic infection, which was associated with increased bacterial persistence. This phenotype correlated with reduced phagocytic activity of hemocytes in both *in vivo* and *in vitro* assays. In contrast, deficiency of Octβ1R or Octβ2R led to enhanced induction of a subset of essential AMP genes upon infection, indicating that octopamine signaling dampens specific humoral immune outputs.

**Conclusion:**

Our data demonstrate that octopamine exerts a decisive influence on the performance of the Drosophila innate immune system by differentially modulating cellular (phagocytosis) and humoral (AMP expression) immune modules in a receptor‑specific manner. These findings establish octopaminergic signaling through Octβ1R and Octβ2R as an important node of neuro‑immune regulation in invertebrates.

## 1 Introduction

Stress responses are essential for helping organisms adapt to environmental challenges, but they also harbor considerable pathological potential. Stress-induced dysregulation can manifest as heightened inflammation, altered immune responses, and disturbances in the hypothalamic–pituitary–adrenal (HPA) axis (1–3). These maladaptations are implicated in a wide array of clinical conditions, including cardiovascular diseases and exacerbations of chronic lung disorders (2, 4, 5).

Although the connection between stress and immune function is well established, the underlying cellular and molecular mechanisms remain incompletely understood. The complexity arises from the diverse nature of stressors (5), their intensities, and the multitude of immune pathways they can modulate (6). Depending on the context, stress may exert immunoenhancing, immunopathological, or immunoregulatory effects (7).

One of the most prominent stress responses is the ‘fight-or-flight’ reaction, which allows rapid physiological adaptation to threats (8, 9). This adaptive process involves coordinated changes in sensory systems, neural pathways, and peripheral organs. In vertebrates, catecholamines such as epinephrine (E) and norepinephrine (NE), secreted by the neuroendocrine system, mediate many of these changes (10). Beyond their systemic effects, catecholamines also modulate immune cell function (11, 12). Importantly, the effects of E and NE on immune cells are highly context-dependent (13–16). These complex responses are mediated through G-protein-coupled α- and β-adrenergic receptors expressed on immune cells (17, 18).

The fight-or-flight response is phylogenetically ancient and is conserved across arthropods, mollusks, and chordates (19, 20). In invertebrates, structurally similar biogenic amines—octopamine (OA) and tyramine (TA)—assume the role of vertebrate catecholamines (21–26). The immune-modulatory effects of OA are context-specific across species. For example, elevated OA levels enhance hemocyte phagocytic activity in the rice stem borer *Chilo suppressalis*, while lower OA levels suppress it (27). Similar effects have been reported in the cockroach *Periplaneta americana* (28) and the armyworm *Spodoptera exigua* (29), Conversely, OA appears to increase susceptibility to infection in the cricket *Gryllus texensis* (19, 30).

In *Drosophila melanogaster*, OA is the primary stress hormone (22, 23). Although some initial studies suggest its involvement in immune modulation (31), the precise roles of OA and its receptors in *Drosophila* immunity remain incompletely understood. OA signals through five known receptors—three classified as β-adrenergic-like and two as α-adrenergic-like (23, 32). A sixth receptor, the Oct-TyrR, responds to both OA and TA (33).

In this study, we focus on the β-adrenergic-like receptors Octβ1R and Octβ2R to dissect their role in innate immune modulation in *Drosophila*. By investigating how these receptors influence immune cell function, we aim to shed light on the evolutionarily conserved mechanisms through which stress hormones shape immunity at the cellular level.

## 2 Results

### 2.1 Expression of octopamine receptor genes in *Drosophila* hemocytes

**Figure 1:**
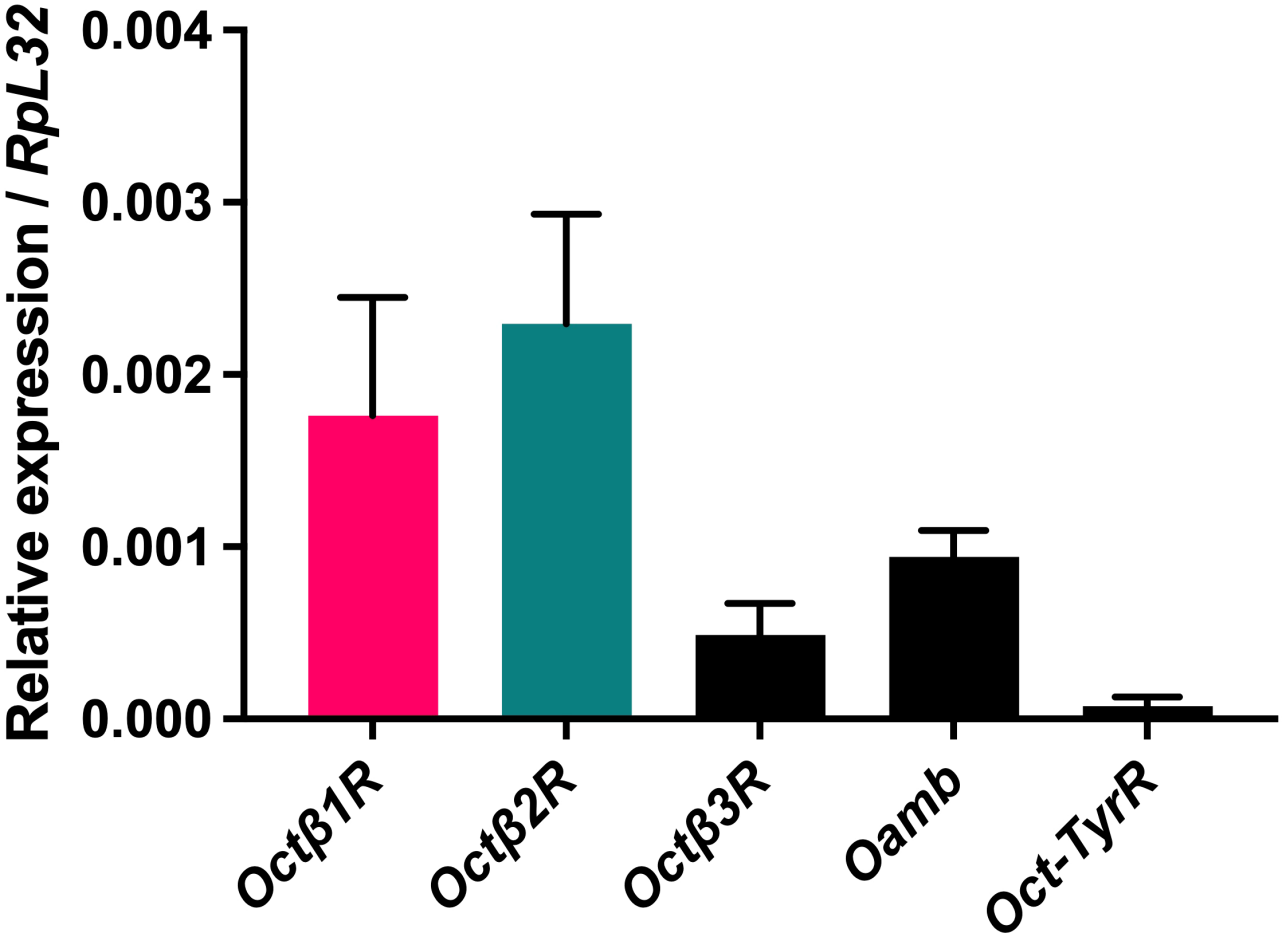
Relative expression of alpha- and beta-adrenergic octopamine receptor genes Octβ1R, Octβ2R, and Octb3R, Oamb, and Oct-TyrR in w^1118^ imagos. The two octopamine receptors Octβ1R and Octβ2R are most strongly expressed in whole flies. Shown is the mean expression ± SEM from nine independent experiments in relation to the housekeeping gene RpL32.

We first employed single-cell data of these cells to elucidate the expression of OA receptors in *Drosophila* hemocytes and fat body cells (34, 35). We found no or only weak signals for the OA receptors of interest. Thus, we looked at general receptor expression in adult flies using conventional qRT-PCR, except for the Octa2R. The highest expression levels were observed for the two b-adrenergic receptor genes *Octβ1R and Octβ2R* (**Figure 1**). For this reason, we focused on the b-adrenergic receptors Octβ1R and Octβ2R and their role in regulating the innate immune response in *Drosophila*.

### 2.2 *Octβ1R* and *Octβ2R* deficient female flies show increased susceptibility to bacterial infection

**Figure 2:**
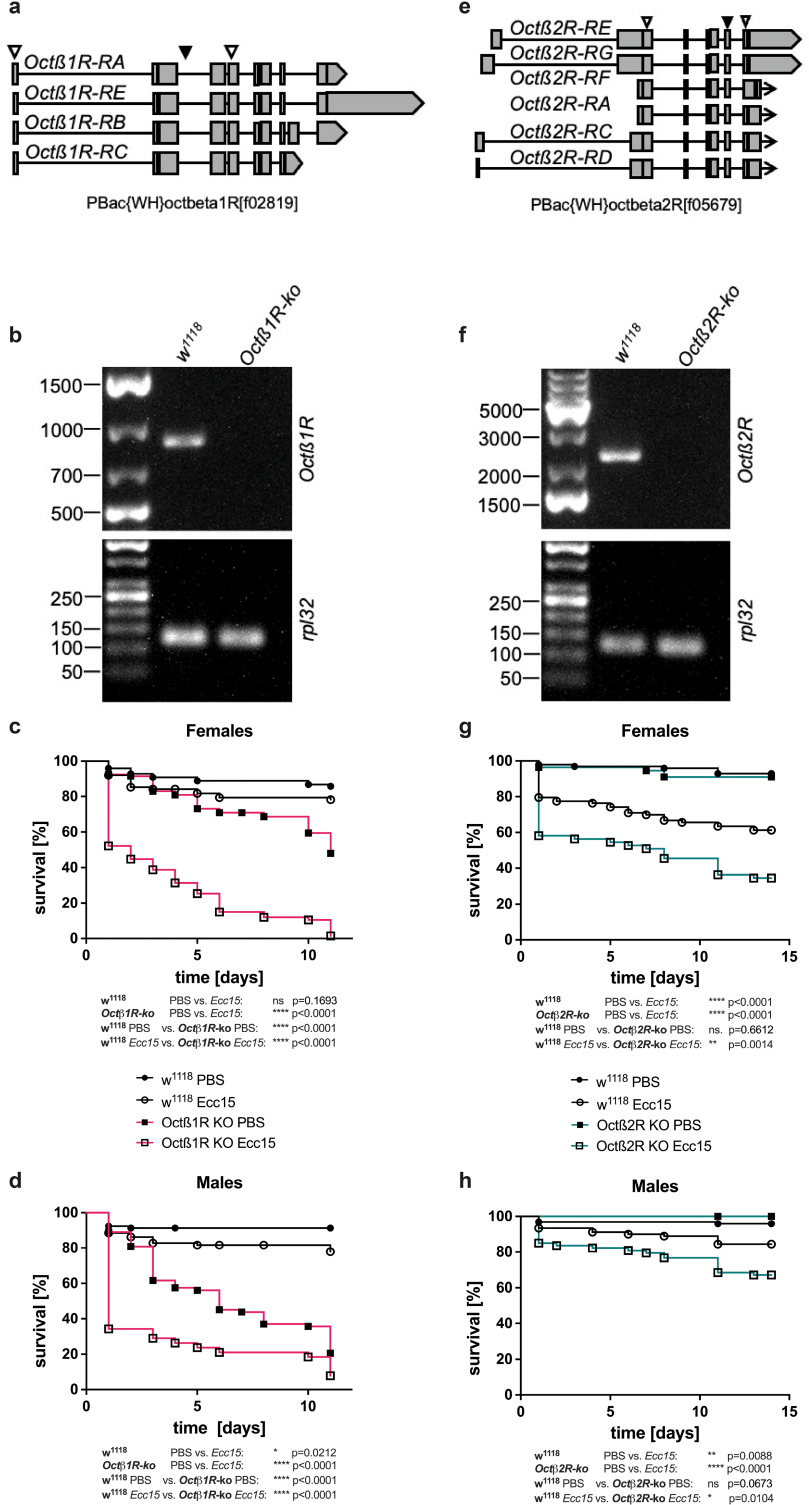
Octopamine receptor knockout strains *Octβ1R*, *Octβ2R*, and their survival after infection with Ecc15. **(a, e)** Insertion sites for the Pbac vectors (black arrowhead) and PCR primer binding sites (white arrowheads) comprise all genetic isoforms of *Octβ1R* or *Octβ2R* genes in the knockout strains. The total knockout of the *Octβ1R***(b)** or *Octβ2R***(f)** gene in the knockout strains has been confirmed by RT-PCR. The RT-PCR products of whole fly cDNA were visualized on a 1% agarose gel. Shown is one generic experiment out of three. Rpl32 was used as a control gene. The survival of *Octβ1R*-ko, *Octβ2R-*ko, and w^1118^ female **(c, g)** and male **(d, h)** flies after infection with *Ecc15* or PBS treatment as a control has been determined. The survival of infected KO females and males is markedly reduced compared to the control flies. Shown are the first 15 days after infection for one of the two generic experiments. The average number of flies in each group was 80 (range 38-99). Statistics: Log-rank (Mantel-Cox) test.

To address the question of the connection between OA and the fly’s immune response, we examined the ability of OA-receptor-deficient flies to fight a bacterial infection. Living bacteria of the Gram-negative strain *Pectobacterium carotovorum* (*Ecc15*) were injected (13.8 nl) into adult flies deficient for the *Octβ1R* or the *Octβ2R* receptor and in flies of the matching genetic control strain w^1118^. *Ecc15* is a plant pathogen that *Drosophila melanogaster* frequently encounters under natural conditions (36) and is often used as the model bacterium for studying bacterial infection in *Drosophila* (37). We injected *Ecc15* in a concentration that activates the animal’s immune response and induces a non-lethal infection. For the infection experiments, *Octβ1R* and *Octβ2R* knockout flies were used, which, due to a directed insertion (**Figures 2a, e**), are unable to transcribe any of the four *Octβ1R* (**Figures 2a, b**) or the six *Octβ2R* (**Figures 2e, f**) transcript variants. Male and female flies were treated separately, and their survival was observed for 15 days. However, only the first eleven days were considered for the statistical analyses (**Figures 2c, d, g, h**).

Two significant results could be drawn from the survival experiments. On the one hand, injection of PBS as a control treatment did not affect female or male survival in the w^1118^ control strain, but it otherwise increased the mortality of *Octβ1R*-ko flies of both sexes (**Figures 2c, d**). Within 5d after injection, 25 % of female and up to 40 % of male *Octβ1R*-ko flies had died. On the other hand, an injection with *Ecc15* led to 15-20% mortality in control females and control males. While more than 70 % of *Octβ1R*-ko females had died after 5 d post-infection, more than 75% of the *Octβ1R*-ko males had died. Taken together, a comparison of the infection’s effect on the survival of female *Octβ1R*-ko vs. control flies indicates that significantly more knockout flies died from the infection. At the same time, the *Octβ1R*-ko animals showed a higher susceptibility to sterile injury.

In contrast, *Octβ2R* knock-out flies did not differ from the w^1118^ flies concerning their response to the sterile injury treatment (**Figures 2g, h**). Neither *Octβ2R*-ko females nor males showed increased mortality after PBS injection. However, the bacterial infection caused a significantly higher death rate of more than 4% at 5 d post-infection in the female *Octβ2R*-ko population compared to 22% of the w^1118^ control animals (**Figure 2g**). In infected *Octβ2R*-ko males, about 20% were dead 5 days post-infection, whereas less than 10% of the matching controls were dead. These differences were highly statistically significant (**Figure 2h**).

To rule out genetic background effects, we analyzed an additional dopamine receptor line generated using the same method as the two octopamine receptor deletion lines. This revealed no differences in survival rates between males and females after infection. These results suggest that differences in general genetic background cannot explain the observed differences in infection response between the two octopamine receptor deletion lines (**Supplementary Figure S1**). Furthermore, we analyzed the involvement of the primary immune tissues, fat, and hemocytes (38), using specific RNAi lines driven into the respective target tissues by either the fat body-specific Cg driver or the hemocyte-specific *Hml* driver. For control experiments, a scrambled RNAi was driven that did not target a specific gene (**Supplementary Figures S2a, b**). Specific knockdown of *Octβ1R* in the fat body significantly reduced resistance to infection compared to the corresponding control. This finding holds for both females (**Supplementary Figure S2a**) and males (**Supplementary Figure S2b**). For *Octβ2R*, we found relatively small effects that were not statistically significant (females, **Supplementary Figure S2c**; males, **Supplementary Figure S2d**). RNAi-mediated silencing in hemocytes showed no apparent effect for *Octβ1R* in females (**Supplementary Figure S2e**) and males (**Supplementary Figure S2f**). Hemocyte-specific silencing of *Octβ2R* resulted in significantly higher mortality in females only (**Supplementary Figures S2g, h**).

### 2.3 *Octβ1R* and *Octβ2R* deficient flies have higher bacterial colonization after systemic infection

We observed a clear correlation between female flies’ higher susceptibility to bacterial infection and their deficiency in the OA receptors Octβ1R and Octβ2R, so we focused on female *Drosophila* for the following experiments.

**Figure 3:**
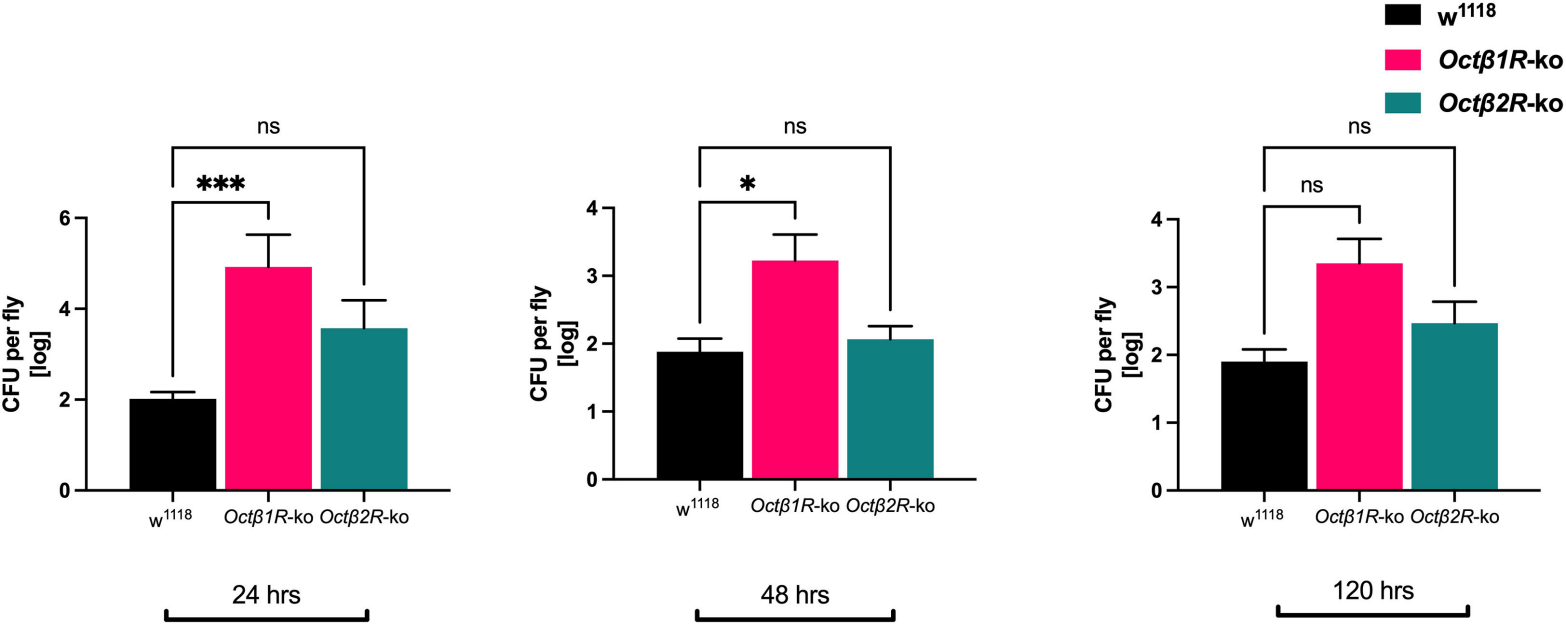
Bacterial load in w^1118^, *Octβ1R*-ko, and *Octβ2R*-ko flies after infection with Ecc15. Adult female flies were infected with *Ecc15* and incubated. 24, 48, and 120 h after injection, six flies per group were analyzed individually, and the amount of living *Ecc15* per animal was determined and is presented as average colony-forming units (cfu) per fly. 24 h after infection, the bacterial load in KO-flies is much higher compared to wildtype flies. For the *Octβ1R*-ko it stays elevated 48 h after infection. Shown are the log-transformed means ± SEM of one out of two generic experiments. Statistics: 1-way ANOVA (correction for multiple testing: Tukey, *p < 0.05; ***p < 0.001).

To gain a deeper insight into why *Octβ1R*- and *Octβ2R-ko* flies show higher mortality after bacterial, we compared their capability to clear bacteria by determining the bacterial load 24, 48, and 120 hours after injection of *Ecc15* (**Figure 3**). A higher bacterial load was detected in *Octβ1R*- and *Octβ2R*-ko flies in comparison to w^1118^ females 24 hours post-infection. While the bacterial load in w^1118^ flies remained at values between 300 and 2000 colony-forming units (cfu) throughout the experiment, the bacterial load in *Octβ1R* and *Octβ2R* ko flies decreased over time. While *Octβ1R*-ko flies showed significantly elevated bacterial loads in all three time points, the cfu number in *Octβ2R-*ko flies was reduced after 48 hours to the level seen in w^1118^ flies.

### 2.4 *Octβ1R* and *Octβ2R* ko flies react to *Ecc15* infection with an elevated expression of AMP genes in comparison to control flies

The immune response of *Drosophila* comprises humoral and cellular components. The fat body mediates the humoral immune response, and the hemocytes mediate the cellular immune response; both cell types are the primary producers of antimicrobial peptides (AMP) (26, 38).

**Figure 4:**
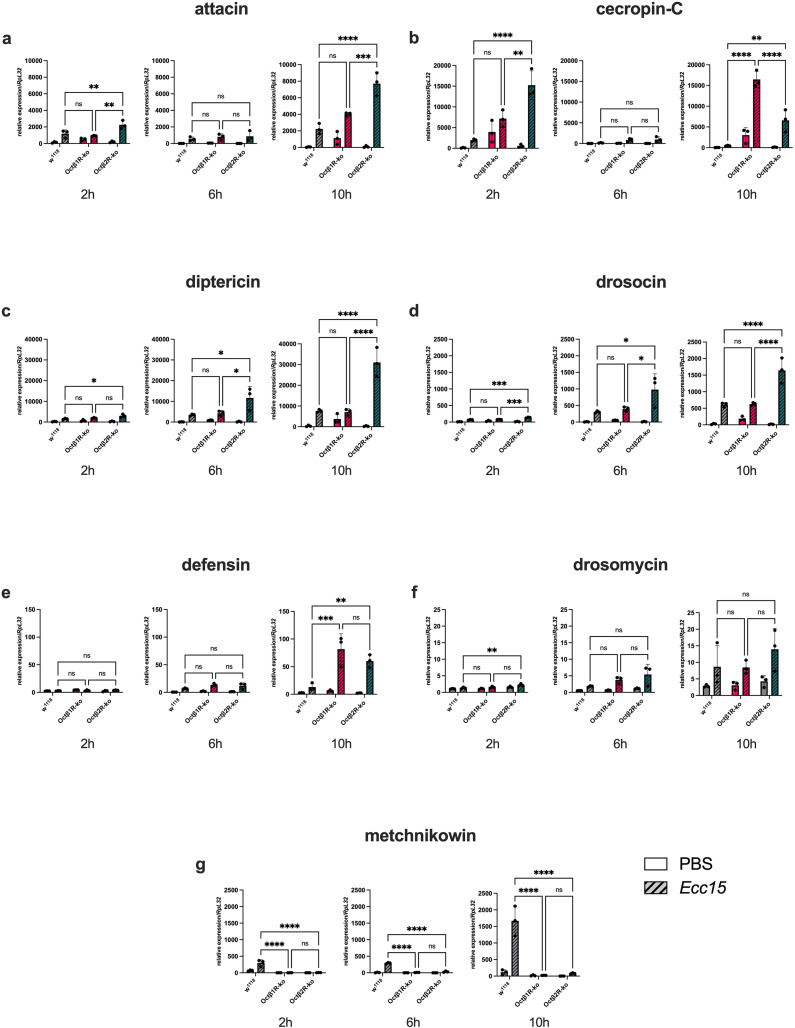
Relative expression of AMP genes after infection with Ecc15. RNA was isolated from w^1118^, *Octβ1R*-ko, or *Octβ2R*-ko six-day-old female flies (3–4 flies per condition), which were either treated with PBS or Ecc15 and incubated for 2, 6, or 10 h. Many AMP genes are strongly upregulated in the KO flies compared to the wild-type flies upon infection. The mean expression of all AMP genes **(a-g)** ± SEM from three independent experiments is shown in relation to RpL32. Statistics: 2-way ANOVA with Tukey multiple comparison test. *p < 0.05; **p < 0.01; ***p < 0.001; ****p < 0.0001.

We monitored the expression levels of all seven classes of AMP genes 2, 6, and 10 h after *Ecc15* injection (**Figure 4**). *Ecc15* infection of control (w^1118^) flies induced a surge in the expression of all AMP genes. While six AMPs *Attacin-A, Diptericin, Drosocin, Defensin, Drosomycin, Metchnikowin*, showed a constant increase over time (**Figures 4a, c-g**), *cecropin-C* expression was only up-regulated two hours after infection, and relapsed rapidly (**Figure 4b**). After six hours, as well as ten hours, the expression remained at the level of PBS-treated animals. A first observation for the AMP expression of infected *Octβ1R-* and *Octβ2R*-ko flies was their inability to up-regulate *Metchnikowin* expression compared to control flies (**Figure 4g**). Infected *Octβ1R*-ko flies showed an equally elevated AMP expression for *Diptericin*, *Drosocin*, and *Drosomycin.* In contrast, *Attacin-A*, *Cecropin-C*, and *Defensin* expression were markedly up-regulated, mainly at the later time point (10 h). However, we observed a strong response to *Ecc15* injection across all AMP-gene expression profiles in *Octβ1R*-ko flies, except *Metchnikowin*. *Octβ2R*-ko animals showed an up-regulation of AMP expression with a time-dependent increase, as observed in w^1118^ control flies. However, in contrast, in control animals, the gain in AMP expression in *Octβ2R*-ko flies was manifold higher after six hours and especially ten hours post-infection (**Figures 4a, c-g**). Again, the expression profile of *Cecropin-C* remained an exception (**Figure 4b**). *Octβ2R*-ko flies also reacted with induction of *Cecropin-C* expression after two hours, however, manifold stronger than control flies. Likewise, this expression peak was followed by a decrease almost to the PBS control level, as observed in w^1118^ flies. However, in contrast, *Octβ2R*-ko responded with a repeated boost in *Cecropin-C* expression after ten hours.

### 2.5 Hemocytes of *Octβ1R* and *Octβ2R* deficient adults show an attenuated phagocytic capacity *in vivo*

**Figure 5:**
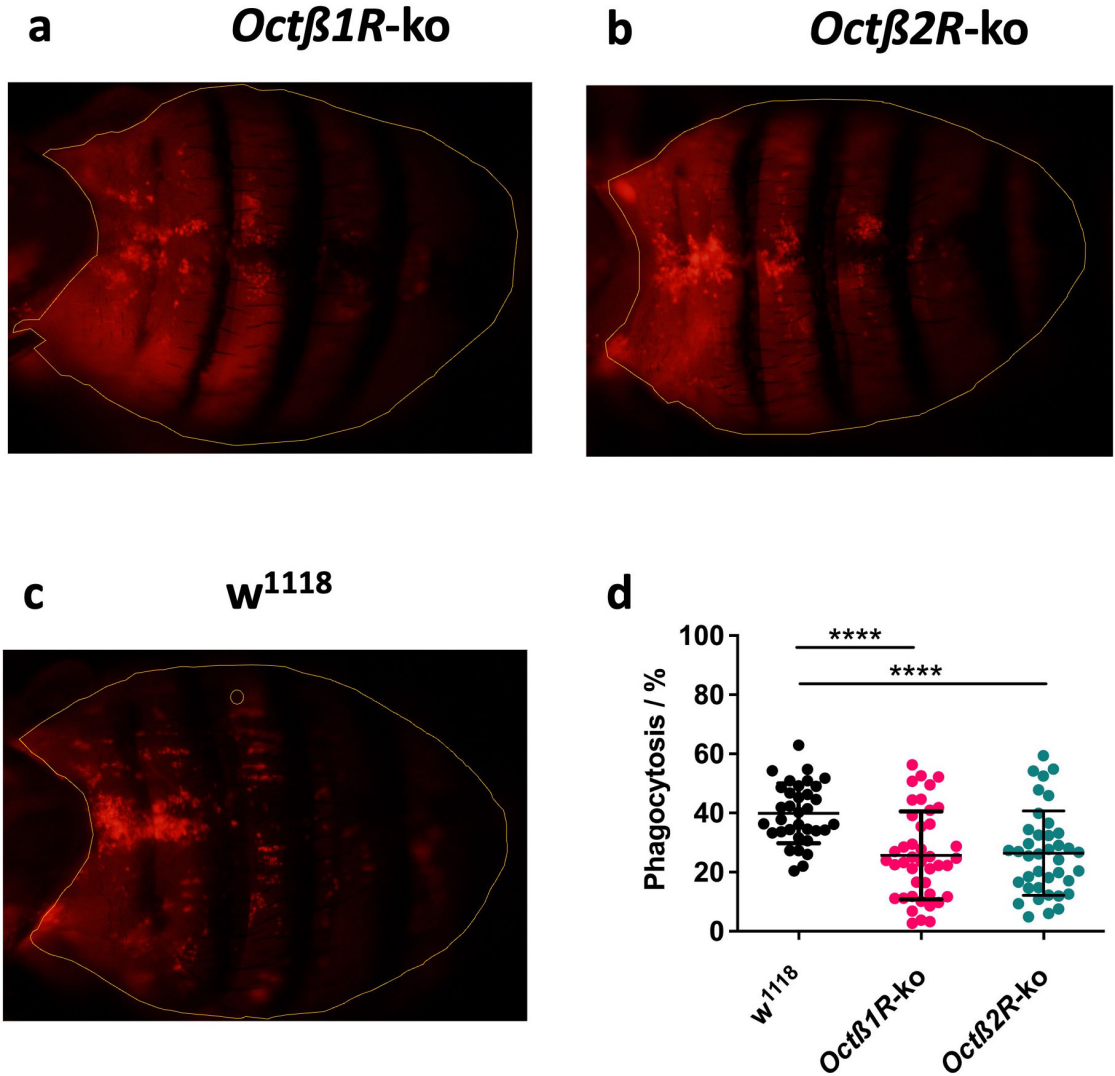
Phagocytic activity of hemocytes in adult flies. Visualization of abdomen 30 min after injection of pHrodo red f. coli bioparticles in six-day old female *Octβ1R*-ko **(a)**, *Octβ2R*-ko **(b)** or w^1118^**(c)** flies. The fluorescence intensity of the particles increases as soon as they are internalized by hemocytes and encounter a pH decline within phagosomes. The yellow rim marks the analyzed abdominal area. **(d)** Quantification of phagocytic activity of hemocytes after internalization of pHrodo bioparticles. Since background fluorescence intensities vary, a threshold has been set, and fluorescence above it has been quantified. The phagocytic activity of hemocytes is reduced in both KO strains compared to the control strain. Shown are the median values ± min to max from at least thirty individuals out of three independent experiments. ****p < 0.0001.

Next, we investigated the impact of Octβ2R and Octβ2R receptors on the cellular components of the immune response. Therefore, we determined the phagocytic activity of hemocytes in response to invading pathogens. To visualize the phagocytic capacity of hemocytes *in vivo*, pHrodo red *E. coli* bioparticles, in place of the Gram-negative *Ecc15* were injected into six-day-old female flies. The fluorescence intensity of pHrodo bioparticles increases dramatically when internalized into the phagosomes and encountering a low pH environment. Hence, the fluorescence intensity within the animal is directly proportional to the amount of phagocytosed bioparticles. Within 30 min after injection, the particles had accumulated within the hemocytes. Because adult *Drosophila* reveals a near-surface concentration of hemocytes in the region of the dorsal abdomen, this area was used for *in vivo* fluorescence intensity quantification (**Figure 5**). Microscopic top views of anesthetized *Octβ2R* - and *Octβ2R*-ko flies already indicated lower particle-borne fluorescence intensity in comparison to control (w^1118^) flies (**Figure 5c**). Concurrently, *Octβ2R-* and *Octβ2R*-ko flies showed a higher background signal (**Figures 5a, b**). Hence, we determined the fluorescence intensity above an individual background threshold (for more information, see **Supplementary Data**). The quantification of the fluorescence intensity above the background threshold confirmed the observation of reduced phagocytic activity in *Octβ2R*- and *Octβ2R*-ko hemocytes (**Figure 5d**).

### 2.6 Larval hemocytes of *Octβ1R* and *Octβ2R*-ko flies show an attenuated phagocytic capacity in *ex vivo* analyses

**Figure 6:**
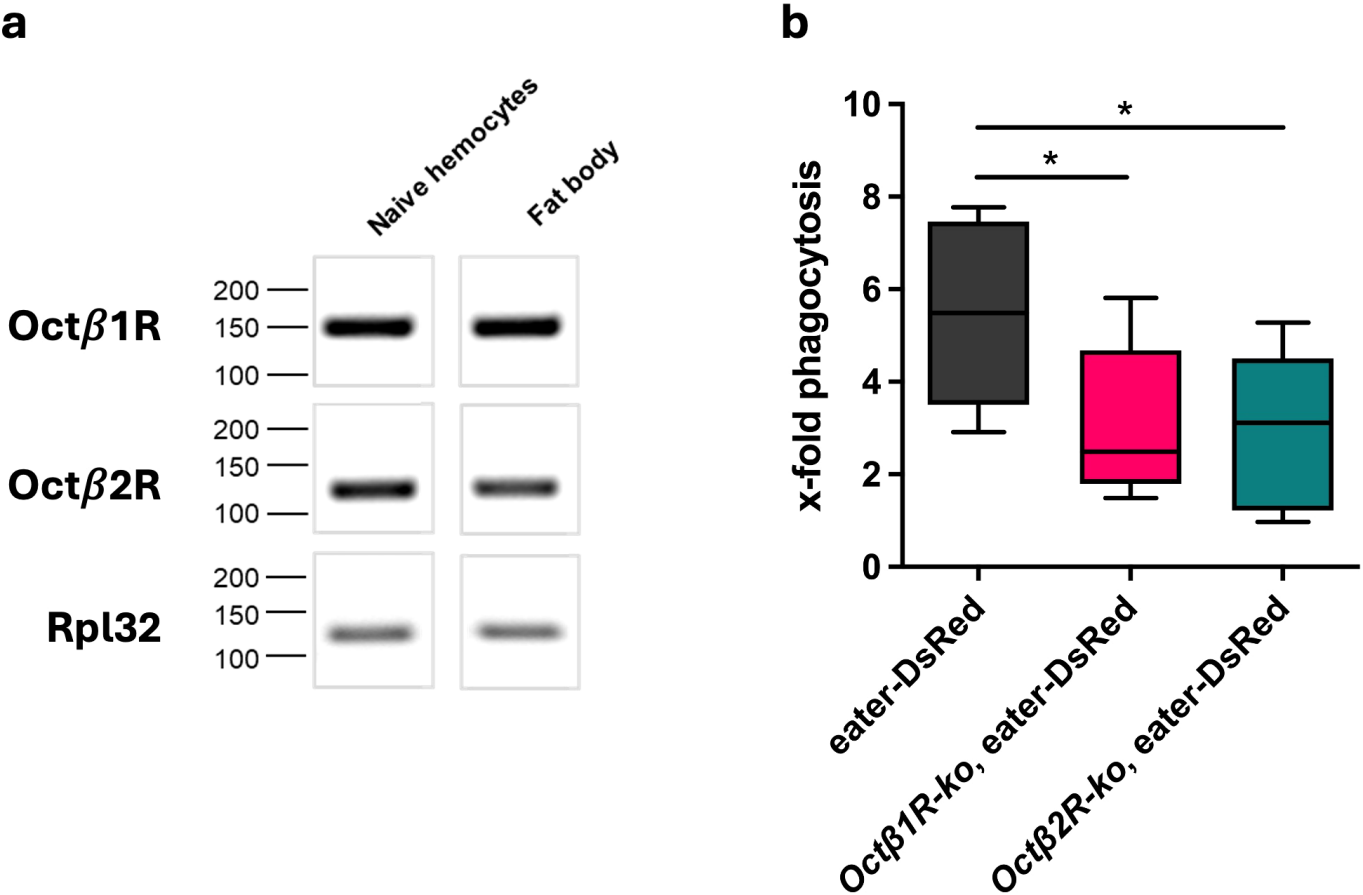
Expression of *Octβ*1R and *Octβ*2R in hemocytes and fat body, and phagocytic activity in hemocytes of stage 3 larvae. **(a)** Hemocytes and fat body were isolated from the larval progeny of a crossbreed of w^1118^ with hml-GAL4, UAS-GFP. Hemocytes were FACS-sorted before RNA isolation. The qRT-PCR products were separated on a gel. *Octβ*1R and *Octβ*2R are both expressed in larval hemocytes and fat body. Shown is one generic experiment out of three. Rpl32 was used as a reference gene for the qRT-PCR analysis. **(b)** Stage 3 larvae were incubated for 30 min after injection of pHrodo E. coli green bioparticles at 25°C or at 4°C. Hemocytes from three larvae per group were isolated and pooled, and the fluorescence intensity of bioparticles was quantified by flow cytometry. The fluorescence intensity of internalized bioparticles at 25°C is normalized to the corresponding 4°C control. Phagocytosis is reduced in both KO strains compared to the control. Shown are means ± SEM from three independent experiments. *p < 0.05.

We also tested the phagocytic capacity of hemocytes isolated from the animal to focus on the circulating hemocyte population in the fly’s body cavity, which might not be reflected in the microscopic technique used in the previous experiment. Beforehand, we confirmed OA receptor gene expression in larval immune tissues, namely the hemocytes and the fat body (**Figure 6a**). According to *in vivo* phagocytosis quantification in adults, pHrodo green *E. coli* bioparticles were injected into 3^rd^ instar larvae carrying a DsRed transgene under the transcriptional control of the eater promoter, together with either an *Octβ1R* or *Octβ2R* deficiency. The eater-DsRed construct emits red fluorescence exclusively from plasmatocytes (39), the phagocytic hemocyte population in *Drosophila*. It facilitated the identification of phagocytes in the flow cytometric analysis of the phagocytosis rate (**Figure 6b**). Compared to hemocytes from eater-DsRed larvae, which served as controls, both *Octβ1R*- and *Octβ2R-*deficient hemocytes showed a significantly reduced phagocytic activity.

To further elucidate the underlying mechanism, we specifically manipulated cAMP levels in hemocytes in *ex vivo* experiments. We used an optogenetic system based on the bacterial light-inducible adenylate cyclase (bPAC (40), which induces cAMP synthesis upon exposure to blue light. Therewith, we mimicked the application of octopamine and the subsequent activation of specific octopamine receptors such as Octβ2R. Here, the UAS-bPACII line was crossed with the hml-Gal4 line. After irradiation and the resulting increase in cAMP, the phagocytic activity of the isolated hemocytes was quantified. The rate of phagocytosis was statistically significantly lower after irradiation (**Supplementary Figure S3a**). In addition, the hemocytes showed increased spreading (**Supplementary Figures S3b, c**), which may be associated with increased substrate binding and could also be quantified (**Supplementary Figure S3b**).

## 3 Discussion

Stress, a multifaceted factor, significantly impacts different aspects of the immune response. The experiments described in this study supported this broad understanding. A key finding of this study, the reduced survival of *Octβ1R*- or *Octβ2R*-deficient flies, underscores the indispensable role of octopaminergic signaling in robust immune defense. Similarly, the higher bacterial levels in receptor-deficient animals provide compelling evidence of the vital role of these receptors. This OA effect is not an isolated case in this study but aligns with findings in other invertebrates and vertebrates. In vertebrates, NE acts via ß-adrenergic receptors on immune cells of the innate and adaptive immune system to stimulate immune responses (41). In invertebrates, we see similar effects caused by the functional equivalent of NE, OA, triggering an immunostimulatory response (29, 42). The immunostimulatory and immunosuppressive effects observed in both groups of organisms may result from differences in the concentrations of these stress-induced ligands. This assumption is supported by findings showing that NE also has immunosuppressive activities, meaning that NE can reduce pro-inflammatory and foster anti-inflammatory signals (43). Although it may be an oversimplification, a potential explanation for this inhibitory effect could be a shift in resource allocation during a fight-or-flight response (44).

We wanted to better understand the influences of this complex hormonal system on the immune response. Thus, we have deliberately focused on the receptor level because it is almost impossible to inhibit octopamine synthesis without unwanted side effects. The only available strategy is to block or inhibit the terminal enzyme in OA biosynthesis, tyramine β-hydroxylase (45, 46). However, this depletes octopamine and massively increases the concentration of the precursor tyramine, which is problematic because tyramine itself has a hormonal role that partially counteracts the octopamine function (23, 25). Our chosen strategy has been widely used and allows a more precise analysis of the hormone receptor system (32, 46, 47). There may be other explanations for the increased expression of AMP genes in animals lacking receptors. The receptor deficiency could directly influence bacterial colonization, thereby inducing this secondary response. However, we have no experimental evidence to support this alternative explanation.

A significant result of the present study is the complex, seemingly opposite effect mediated by the OA receptors Octβ1R and Octβ2R across different facets of the immune response. Both octopamine receptors belong to the same receptor family and presumably act through cAMP signaling. Despite these similarities, Octβ2R acts through Gs, leading to cAMP production, while Octβ1R might also have inhibitory activities mediated through Go, at least under certain conditions (48). This specific type of effectuation might result in antagonistic activity of both receptors under certain conditions. Together with differences in expression patterns of both receptors, this might explain the different effects observed here. Thus, compensation for the lack of receptors by other OA receptors is possible, but not probable. Our observation that the corresponding deficient animals show higher induced levels of AMPs after bacterial challenge implies an inhibitory role of OA for the level of AMP expression. Most of the AMP amount observed after an immune challenge of *Drosophila* is produced by the fat body, which is the main organ relevant to the humoral immune response of insects (26, 38). This holds for major AMPs, including Attacin-C, Cecropin-C, Drosocin, Drosomycin, Defensin, and Diptericin. One of these major AMP genes, *Metchnikowin (Mtk)*, shows the opposite behavior, further complicating this picture. *Mtk* is an AMP that is highly effective against a broad range of fungi and bacteria, and it is a relevant part of *Drosophila*’s immune response (49, 50). It is distinguished from other AMPs by the presence of distinct alleles with substantially divergent functionalities (49). Moreover, it has a neuronal function that becomes apparent under highly stressful conditions (51). These *Mtk* alleles, differentiated by only one amino acid, are maintained in the population. Under certain conditions, the presence of *Mtk* expression is even deleterious (49). Thus, the observed effects of the octopaminergic signaling system might specifically interfere with induced Mtk expression, adding another level of complexity to the regulatory system for this specific AMP. The interpretation that for the other relevant AMPs, OA exerts adverse effects means that octopaminergic stress signaling dampens this type of immune response substantially, which is in line with at least several reports.

Besides this effect on humoral immunity, our results also show a regulation of cellular immunity. Phagocytosis of bacteria also depends on the activity of the octopaminergic system, which is a valid interpretation, as the two OA receptor-deficient fly lines we used show a reduced phagocytotic activity of hemocytes. This effect was seen *in vivo* and *in vitro*. Thus, it can be assumed that OA increases phagocytotic activity and, in addition to that, has a positive effect on this arm of the innate immune system of the fly. This agrees with recent findings (28, 29, 52) showing a stimulatory role of OA on phagocytosis in hemocytes. Looking at the role of NE in phagocytosis, the situation is more complex as stimulatory and inhibitory effects were reported. NE stimulated, for example, phagocytotic activity of macrophages (53, 54), whereas phagocytosis by neutrophils appears to be inhibited, at least in the context of wound healing (55). Moreover, the effects of OA, like the effects of NE in vertebrates, seem to be concentration-dependent, showing opposite effects at different concentrations, which makes it more complicated to obtain a clear picture of the physiological role of these stress hormones for immune regulation (24, 42).

Our RNAi experiments targeting either the fat body or the hemocytes support the role of both receptors as mediators of an immunomodulatory action in both tissues. We observed reduced survival in flies depleted for the receptors in both tissues. This was predominantly observed in females, whereas for males, we observed a more complex picture. These results thus support our interpretation that both OA receptors modulate both immune-competent organs.

OA and TA are generally considered to be the invertebrate equivalents of E and NE. This is due not only to their very similar structures, but also to the fact that both systems perform similar functions within their respective organism groups (32). However, the attribution at the receptor level is more difficult and less clear-cut. Although alpha and beta octopamine receptors have been clearly identified in *Drosophila*, it is more challenging to make these connections (23). Consequently, there remains a degree of uncertainty when transferring these results to humans.

An essential aspect of this study is the significant sex specificity of the immune response. This relates to general differences in susceptibility to infection and the increased vulnerability of *Octβ1R* -deficient females to septic infections, a response not observed in males. Other reports have shown differences in immune responses between the sexes, with males investing more in combating gram-negative bacterial infections (56). This aligns with the results of the current study, as the infection was performed with the gram-negative bacterium *Pectobacterium carotovorum (Ecc15)*. The ability of female flies to adjust their response to an infection comes at the cost of reduced survival under certain conditions (57). Differences in immune performance between sexes appear to be a common phenomenon rather than an exception (58–60). Furthermore, differences in immune performance between sexes appear to be a common phenomenon rather than an exception (58–60). Generally, differences between the sexes that lead to different outcomes of an infection may also depend on the distinct metabolic properties of males and females. Females have higher food intake and, consequently, an increased metabolic rate, leading to differences in other energy-requiring activities, such as the immune response, between the sexes (61, 62).

This study’s critical insight is the differential regulation of the two major arms of the innate immune system, the humoral and the cellular immune responses. Both arms can be associated with different immune-competent organs of the fly, the fat body as the primary source of the humoral immune response, and the hemocytes as the cellular substrate of the cellular response. OA shifts investment in the immune system from the fat body to the hemocytes, which may represent a strategic reallocation of resources to enable survival during intense stress. Besides its effect on the immune system, stress, mediated by OA, also shifts the body’s metabolism and energy use, which is primarily located in the fat body (63). OA disentangles the dual functions of the fat body, fueling the humoral immune response and supplying the organism with sufficient energy. This appears necessary as the fat body is prone to show functional impairment if challenged strongly. A similar conflict was recently demonstrated for immune responses and reproduction (64). In our system, OA is an ideal candidate to solve this type of conflict by shifting the activity of the immune system’s humoral, fat body-based arm towards the cellular arm of the immune response. Taken together, the shift of the immune performance induced by OA signaling might reflect the impact of stress on this important trait that is interconnected with various other traits.

However, when looking at this study, it is crucial to remember that it is the first to characterize this highly relevant phenomenon, leaving room for future studies. Using genomic KO flies, we could show that the two receptors, *Octβ1R* and *Octβ2R*, play essential roles in regulating different facets of the immune defense. Still, a precise assignment to the relevant target organs is difficult despite first RNAi experiments. The central part of the work shows that macrophage-like hemocytes are the relevant target cells for the effects on phagocytosis. Cell type-specific analyses on this issue are reserved for follow-up studies.

## 4 Materials and methods

### 4.1 *Drosophila* strains and husbandry

Fly strains were supplied by the Bloomington *Drosophila* Stock Center (Indiana University, Bloomington, USA) or by colleagues. w^1118^ (BL5905), y^1^w^1118^ (BL6598), *Octβ1R*- (BL18589), *Octβ2R*-deficient (BL18896) and *Dop1R1*-deficient (BL605531) stocks were obtained from the BDSC. The eater-DsRed strain was a kind gift from Laura Vesala (University of Tampere, Tampere, Finland). For FACS sorting of hemocytes and following RNA isolation, the offspring from the cross w^1118^; hml-GAL4, UAS-GFP (BL6397) > w^1118^ (BL5905) was used, hereafter referred to as hml, GFP > w^1118^. RNAi experiments were performed with the Cg-Gal4 (BL 7011) and the Hml-Gal4 (BL 6397) driver lines and the Valium10-control RNAi (BL 35786), as well as those for the *Octβ1R (*BL 58179*)* and *Octβ2R* (B 50580), respectively. For the optogenetic experiments, we used a UAS-bPACII strain provided by Martin Schwärtzel (Berlin, Germany). All strains were kept on standard cornmeal-yeast medium (46) at 21°C at a relative humidity of 60 % in a 12 h:12 h light-dark cycle if not otherwise stated.

### 4.2 RNA isolation and quantitative real-time PCR analysis

For RNA isolation, ten adult flies of each genotype and treatment (PBS or *Ecc15*) were transferred to a 2 mL screw cap microtube and snap-frozen in liquid nitrogen. Five pre-cooled stainless-steel beads (ø 3 mm, IHSD-Klarmann, Bamberg, Germany) were added before flies were homogenized with the Tissue Lyser LT (Qiagen, Hilden, Germany) for 2 min at 25 Hz. Samples were then mixed with 100 µL RA1 lysis buffer (Macherey-Nagel NucleoSpin RNA Kit, Dueren, Germany), homogenized again, and filled up with RA1 buffer to a final volume of 350 µL before freezing them at -80°C until further processing.

Larvae: Disinfected 3^rd^ instar larvae were transferred to 350 µL RA1 lysis buffer and manually homogenized using a Kontes pellet pestle (Fisher Scientific, Schwerte, Germany). RNA isolation from larvae and adult flies was performed according to the manufacturer’s protocols.

After RNA extraction, the remaining genomic DNA was digested by Turbo DNase (Invitrogen Turbo DNA-free Kit, Thermo Fisher Scientific, Vilnius, Lithuania). Total RNA concentration and purity were determined with the nanophotometer P330 (Implen, München, Germany). According to the manufacturer’s instructions, RNA (400 ng if not otherwise stated) was transcribed to cDNA using the SuperScript III First-Strand Synthesis System (Thermo Fisher Scientific, Carlsbad, USA). Quantitative real-time PCR (qRT-PCR) was carried out with 2 µL amplified cDNA in a 10 µL reaction volume of LightCycler 480 SYBR Green I Master Mix according to the manufacturer’s instructions (Roche Diagnostics, Mannheim, Germany). A reaction mixture without cDNA served as a negative control. Transcript levels were measured on a LightCycler 480 II in LightCycler 480 96-Multiwell Plates. Amplification and melt curves were generated by the LightCycler software version 3.5 (Roche Diagnostics, Mannheim, Germany). All samples were analyzed in triplicates, averaged, and normalized against the housekeeping gene *RpL32*. At least three independent biological experiments were performed.

The following primers were used: *Attacin-A (AttA):* sense 5’-GAGGCACTTCCCACAACAGG-3’, antisense 5’-ACCAGCGGGATTGGAGGTTA-3’; *Diptericin (Dpt):* sense 5’-AATGGACGCCACGAGATTGG-3’, antisense 5’- GCTCGGTTCTGAGTTGCCAT-3’; *Drosocin (Dro):* sense 5’-GTTCACCATCGTTTTCCTGC-3’, antisense 5’-GGCAGCTTGAGTCAGGTGAT-3’; *Metchnikowin (Mtk):* sense 5’-TCTTGGAGCGATTTTTCTGG-3’, antisense 5’-TCTGCCAGCACTGATGTAGC-3’; *Drosomycin (Drs):* sense 5’-GAGACCTGTCGTCGTGTGTG-3’, antisense 5’-TTTAGCATCCTTCGCACCA-3’); *Defensin (Def):* sense 5’-TTTTGCTCTGCTTGCTTGC-3’, antisense 5’-ACATGATCCTCTGGAATTGGA-3’; *Cecropin-C (CecC):* sense 5-’GGCAAGAGAATCGAGCGCAT-3’, antisense 5’-GCGCGTTATCCTGGTAGAGT-3’; *ribosomal protein L32 (RpL32)* sense 5’- CCAGTCGGATCGATATGCTAA-3’, antisense 5’-GTTCGATCCGTAACCGATGT-3’; *Octβ1R*: sense 5’-CGGTCGACAGATACTACGCC-3’, antisense 5’-GTTGTGTACCATCCCGAGCA-3’; *Octβ2R*: sense 5’-TCCTGTGGTACACACTCTCCA-3’, antisense 5’- -3’; *Octβ3R*: sense 5’-AAGTACGCGTAGATCAGCGG-3’, antisense 5’- -3’; *Oamb*: sense 5’-CCGGCATTTGGGTACTCTCA-3’, antisense 5’-GTTGACCATCCTCCGAGCTT-3’; *Oct-tyrR*: sense 5’-GAATGCGAAGGAGCTGTGGA-3’, antisense 5’-TGTTCCCGATGATGGTCAGC-3’.

### 4.3 Larval fat body and hemocyte isolation for qRT-PCR analysis

Late 3^rd^ instar larvae (hml,GFP > w^1118^) were washed with PBS, disinfected with NaClO (5 %, dissolved in H_2_O) for two min., rewashed with PBS, and placed on ice for no longer than 30 min. Hemocytes were isolated by bleeding; therefore, larvae were dried on a paper tissue, dipped into a well of a 96-well ultra-low attachment plate (Corning Costar, Merck, Darmstadt, Germany) filled with 330 µl of precooled Schneider’s *Drosophila* medium (Gibco, Thermo Fisher Scientific, Schwerte, Germany) and were carefully opened at the dorsal side by opening the cuticle with a pair of Dumont N°5 biologic forceps. 15 male and 15 female larvae were bled out per well. The contents of each well were transferred to a well of a MultiScreen-Mesh Filter Plate (Merck Millipore, Darmstadt, Germany) with a 40 µm mesh to remove larger particles or tissue residues. Filtrates of six wells (at least hemocytes of 180 larvae) were pooled in a FACS tube and sorted by a FACS Aria (BD Biosciences, San Jose, USA) equipped with a 488 nm laser for excitation of GFP and a bandpass filter (530/30) to detect GFP-positive cells. The purity of sorted fractions was checked by FACS reanalysis. GFP-positive hemocytes were sorted in 200 µL of precooled Schneider’s medium, transferred to a 1.5 mL centrifuge tube, and pelleted by centrifugation at 4°C and 1500 x g for 5 min. The medium was discarded except for a 50 µL residue to prevent accidentally sucking up cells. The cells were resuspended in 100 µL of RLT-buffer (RNeasy Micro Kit, Qiagen, Hilden, Germany), homogenized using a pellet pestle, and 1 µL of β-mercaptoethanol was added before freezing the sample at -80°C until further processing. Six samples of that kind were pooled to obtain one replicate.

For fat body isolation, six male and six female late 3^rd^ instar larvae (hml, GFP > w^1118^) were washed as described above. The fat body was removed with Dumont N°5 forceps and transferred to a 1.5 mL tube with 100 µL RNAprotect Cell Reagent (Qiagen, Hilden, Germany). The sample was pelleted at 4°C and 4500 x g for 5 min. The supernatant was removed, and the sample was homogenized with a pellet pestle in 350 µL of RLT buffer. 3.5 µL of β-mercaptoethanol was added, and the sample was frozen at -80°C until further processing.

Total RNA from hemocytes and the fat body was isolated according to the Micro Kit protocol and subsequently treated and analyzed as described above for RNA samples from adults or larvae. 300 ng of RNA was used for cDNA transcription. qRT-PCR products were applied to a 2.5 % agarose gel stained with ethidium bromide.

### 4.4 Analysis of *Octβ1R* and *Octβ2R* mutants

Reverse transcriptase PCR was performed to verify the PBac(WH) insertions in the *Octβ1R* and *Octβ2R* mutant strains. Therefore, RNA was extracted from adults and transcribed to cDNA, as described above. Primers used to indirectly verify the presence of PBac (WH) Octβ1R [f02819] or PBac(WH)Octβ2R[f05679] were 5’-CCGCCTGGCAACGAGTAAC-3’ (sense) and 5’-GATAGCCACACCATCAATAGC-3’ (antisense) or 5’-CGCGTGTCGTGATTTCCCAA-3’ (sense) and 5’- TTAATGGAACACTTTAA TTGC-3’ (antisense), respectively. These primers were designed to recognize all known splice variants of *Octβ1R* and *Octβ2R*. PCR products were applied on a 2 % agarose gel stained with ethidium bromide. The GeneRuler 1 kb Plus DNA Ladder or 50 bp DNA Ladder (Thermo Fisher Scientific, Vilnius, Lithuania) served as internal standards to determine the accurate fragment size of amplified cDNA.

### 4.5 Infection of adult flies

*Pectobacterium carotovorum Ecc15* (CFBP2141, Cirm, Beaucuzé, France) was cultured in LB-broth overnight (about 14 h) at 29°C. The culture was spun down and resuspended in sterile PBS to achieve an optical density (OD) of 2. Six to seven days old males or virgin female flies were CO_2_ anesthetized and injected intrathoracically (mesopleural region) with either 13.8 nL of *Ecc15* or PBS (mock control) by using a Nanoject II microinjector and glass capillary: 3.5” Drummond: OD = 1.14 mm, ID = 0.53 mm, L = 90 mm; tip pulled: ID = 15 μm +/- 2, L ~ 5.5 mm. (Drummond Scientific/Suedlabor, Gauting, Germany).

For the survival experiment, up to 50 flies per group were kept in fly culture vials (175 mL) supplied with a standard fly medium at 29°C after infection. The medium was exchanged every two days without anesthetizing the flies. Dead flies were counted and removed from the vials daily.

Ten vivid flies per group were collected 2, 6, or 10 h post-injection to quantify AMP gene expression. RNA was isolated and further treated as described above.

### 4.6 Bacterial load

As described above, a rifampicin-resistant strain of *P. carotovorum* (Cirm, Beaucuzé, France) was cultured and prepared for injection. Ten six to seven-day-old female flies were injected with 13.2 nL of rifampicin-resistant *Ecc15* as described above and kept on standard food at 29°C for 24, 48, or 120 h. Six flies of each genotype were then homogenized individually with a Kontes pellet pestle (Fisher Scientific, Schwerte, Germany) in 100 µL of ice-cold PBS. Afterward, 150 µL PBS was added to the lysates before they were serially diluted in PBS, starting with 1:10 followed by four subsequent 1:10 dilutions. Afterward, 10 µL of each dilution were pipetted on LB-agar plates containing 100 µL/mL rifampicin and incubated upside down at 29°C for 18 to 20 h. Finally, the plates were photographed, and bacterial colonies were counted.

### 4.7 *In vivo* phagocytosis assay in *Drosophila* adults

Ten five- to six-day-old female flies of each genotype were anesthetized by CO_2. E_ither 101.2 nL (2 x 50.6 nL) of 2 mg/mL pHrodo red *E. coli* bioparticles (Life Technologies, Eugene, USA) dissolved in HBSS buffer (Gibco, Thermo Fisher Scientific, Schwerte, Germany), supplemented with 20 mM HEPES (pH 7.5), were injected by using a microinjector as described above or HBBS buffer was injected as a mock control. The bioparticles had been sonicated twice for 5 min. and were kept on ice until used. After injection, flies were incubated for 60 min at 25°C before anesthetizing them with the FlyNap Aesthetic Kit (Carolina Biological Supply, Burlington, USA) for 4 min. Wings were removed with Dumont N°5 forceps, and flies were placed on a microscope slide, dorsal side up, and covered with a cover glass. The fluorescently labeled bioparticles were visualized through the abdominal cuticle using a fluorescence stereo microscope (Olympus, Hamburg, Germany) using SDF Planapo 2x PFC with 2.5 x zoom and the fluorescence filter SZX2-FRFP2. Fluorescence images were captured with a DP72 camera and edited using CellSens Standard 1.16 (Olympus, Hamburg, Germany). The relative fluorescence (a measure of phagocytic index) was calculated with ImageJ 2x2.1.4.7 software (National Institutes of Health, Bethesda, USA). The phagocytic index was determined by calculating the ratio of pHrodo signals to total fluorescence. For a more detailed description, see supplemental **Supplementary Figure S4**.

### 4.8 *In vivo* phagocytosis assay in *Drosophila* larvae

Female, late 3^rd^ instar larvae were collected, washed with PBS, and disinfected in 70 % ethanol for 2 min. They were immobilized in 300 µL of PBS containing dichlorvos (1:1000; Sigma-Aldrich, Merck, Darmstadt, Germany) for 5 min at room temperature and washed three times with PBS. The pHrodo green *E. coli* bioparticle solution (2 mg/mL, pH 7.5; Life Technologies, Eugene, USA) was dissolved and treated as described above. Larvae were dried on a paper towel, put on a glass slide, and either 100 nL (2x 50 nL) of bioparticles or HBSS-buffer were injected using a microinjector (Nanoject III, Drummond, Suedlabor, Gauting, Germany). Before use, the glass capillaries (described above) were sharpened by cutting the tip. Bioparticles or HBSS buffer were injected into the hemocoel at the ventral side between larval segments A6 and A7 (30). Then, each larva was carefully placed in a well of a 96-well plate partially filled with glucose-agar medium (5 % glucose solved in 1 % agarose solution) to prevent them from drying out. Larvae were incubated for 30 min at 25°C while corresponding controls were kept on ice. Three larvae were bled into a 20 µL drop of HBSS buffer (supplemented with 20 mM HEPES (pH 6.8)) and placed on a pre-cooled microscope slide. Hemocytes were transferred into a 1.4 mL push cap tube (Micronic, Lelystad, The Netherlands) containing 80 µL of the same buffer. The push cap tubes were placed in standard FACS tubes and kept on ice until samples were analyzed with the LSRII (BD Biosciences, San Jose, USA) flow cytometer. Fluorescence was excited with a 488 nm laser. The DsRed-positive hemocytes were identified by a positive signal in the PE-Texas red channel (BP filter 610/20), and fluorescence intensity of ingested pHrodo green *E. coli* bioparticles was detected in the FITC channel (BP filter 530/30). Analysis was performed at a medium flow rate until 4000 DsRed-positive events were counted. With the help of the FCS express software (vers. 6.05.0028), the cells were first divided into DsRed-positive and -negative populations. Doublets were excluded from the DsRed-positive population using the forward and sideward scatter channels. The geometric mean of the green signal of the remaining population was determined.

For the optogenetic experiments, we used hemocytes from larvae with the genotype (hml-Gal4, UAS-bPACII). Larvae were kept in the dark, and the isolation procedure was performed in the dark as well. For the blue-light activation, we used an approved approach similar to that described (40). Quantification of phagocytosis was performed as previously described. Spreading was calculated as the occupied area.

### 4.9 Statistical analysis

All statistics have been calculated by GraphPad prism v10.6.1. The following tests were used: Log-rank (Mantel-Cox), **Figure 2**; 1way ANOVA with Tukey multiple comparison test, **Figure 3**; 2way ANOVA with Tukey multiple comparison test, **Figure 4**; 1way ANOVA of log-transformed cfu counts with Tukey multiple comparison test, **Figure 5**; 1way ANOVA with Tukey multiple comparison test. Statistical tests are also indicated in each figure legend.

### 4.10 Limitations of the study

The study was performed with genomic deficiencies of the two octopamine receptors of interest. Thus, crosstalk between different organs might also be changed, making it difficult to assign a specific phenotype to a given organ. Moreover, a direct translation of the result to the situation in vertebrates is hard.

## Data Availability

The original contributions presented in the study are included in the article/**Supplementary Material**. Further inquiries can be directed to the corresponding authors.

## Supplementary material

Supplementary Figure 1 Dopamine Dop1R1-deficient strain shows no differences in infection compared to the matching control. The survival of *Dop1R1*-ko, and w^1118^ female **(a)** and male **(b)** flies after infection with *Ecc15* or treatment with PBS as a control has been determined. Shown are the first five days after infection for one of the three generic experiments. Each group contained at least 50 flies. Statistics: Log-rank (Mantel-Cox) test.

Supplementary Figure 2 Octopamine receptor RNAi and their survival after infection with Ecc15. The survival of *Octβ1R*-RNAi **(a-d)**, *Octβ2R-*RNAi (e-h), and their matching controls (RNAi targeted against an unrelated sequence). RNAi of *Octβ1R* driven in the fat body by Cg-Gal4 for females **(a)** and males **(b)**. RNAi of *Octβ2R* driven in the fat body by Cg-Gal4 for females **(c)** and males **(d)**. RNAi of *Octβ1R* driven in the hemocyte by Hml-Gal4 for females **(e)** and males **(f)**. RNAi of *Octβ2R* driven in the hemocytes by Hml-Gal4 for females **(g)** and males **(h)**. Each group contained at least 50 flies. Statistics: Log-rank (Mantel-Cox) test.

Supplementary Figure 3 Induced increase in cAMP in hemocytes reduced phagocytosis while in increased substrate attachment. Blue-light induced increase in cAMP of hml-Gal4, UAS-bPacII induced reduced phagocytosis of ex vivo hemocytes **(a)**. Substrate spreading response in non-irradiated hemocytes **(b)** and those that experienced blue light **(c)**. Quantification of the covered areas of non-irradiated and irradiated hemocytes **(d)**. n ≥ 445, unpaired t-test.

Supplementary Figure 4 Fluorescence images of the adult female abdomen. **(a)**: The background fluorescence was determined in a small area in the third abdominal segment (yellow circle). **(b)**: The total fluorescence amount was ascertained by encircling the entire abdomen (yellow rim). **(c)**: Calculation for the phagocytic index.
