# Supplementary material for: Octopamine modulates the innate immune response in Drosophila melanogaster
Source: Front Immunol. 2026 Jan 8;16:1720126. doi: 10.3389/fimmu.2025.1720126 (PMC12823492; doi:10.3389/fimmu.2025.1720126)
Supplement: Supplementary Figure 1 — Dopamine Dop1R1-deficient strain shows no differences in infection compared to the matching control. The survival of Dop1R1-ko, and w1118 female (a) and male (b) flies after infection with Ecc15 or treatment with PBS as a control has been determined. Shown are the first five days after infection for one of the three generic experiments. Each group contained at least 50 flies. Statistics: Log-rank (Mantel-Cox) test. [file DataSheet1.pdf]

Supplementary Material

Supplementary Figures

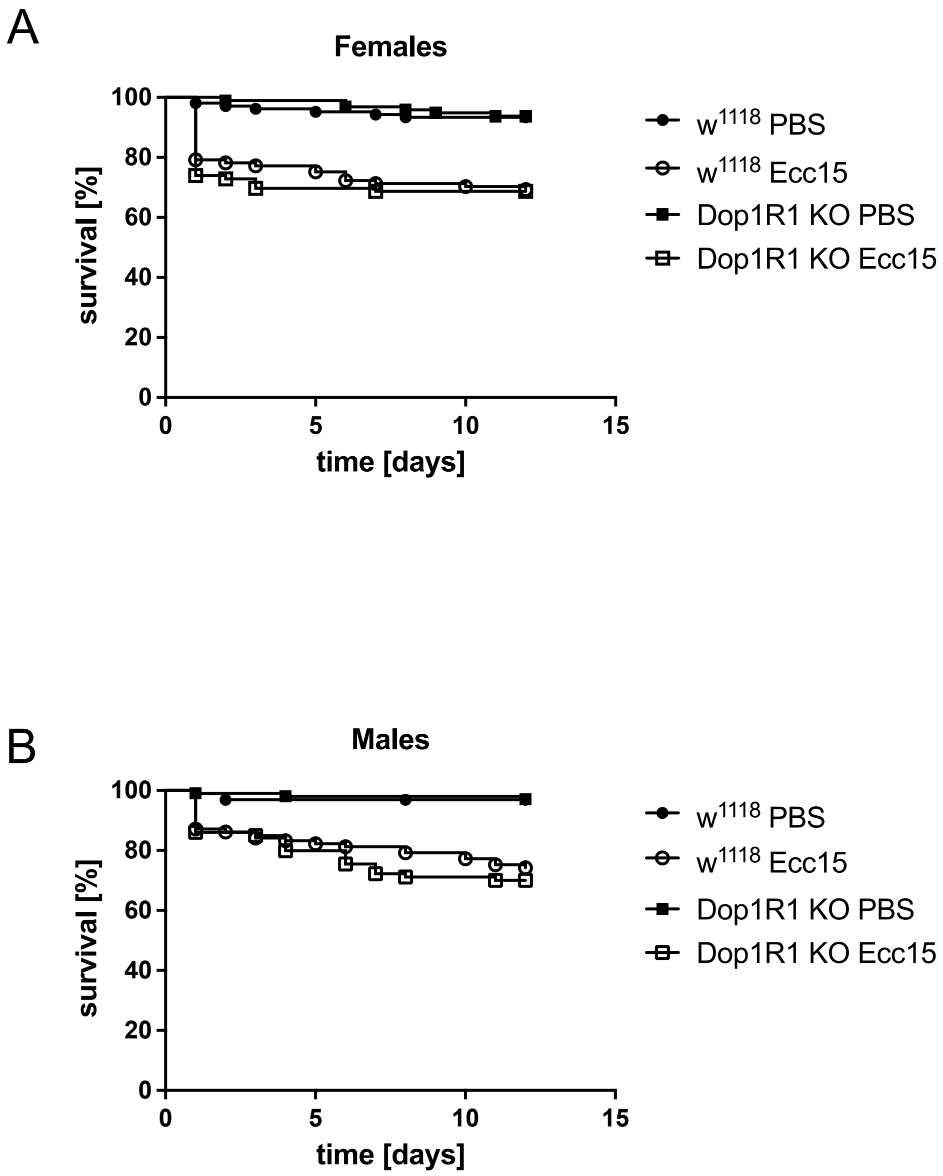

Supplementary Figure 1. Dopamine Dop1R1-deficient strain shows no differences in infection compared to the matching control. The survival of *Dop1R1*-ko, and w^{1118} female (a) and male (b) flies after infection with *Ecc15* or treatment with PBS as a control has been determined. Shown are the first five days after infection for one of the three generic experiments. Each group contained at least 50 flies. Statistics: Log-rank (Mantel-Cox) test.

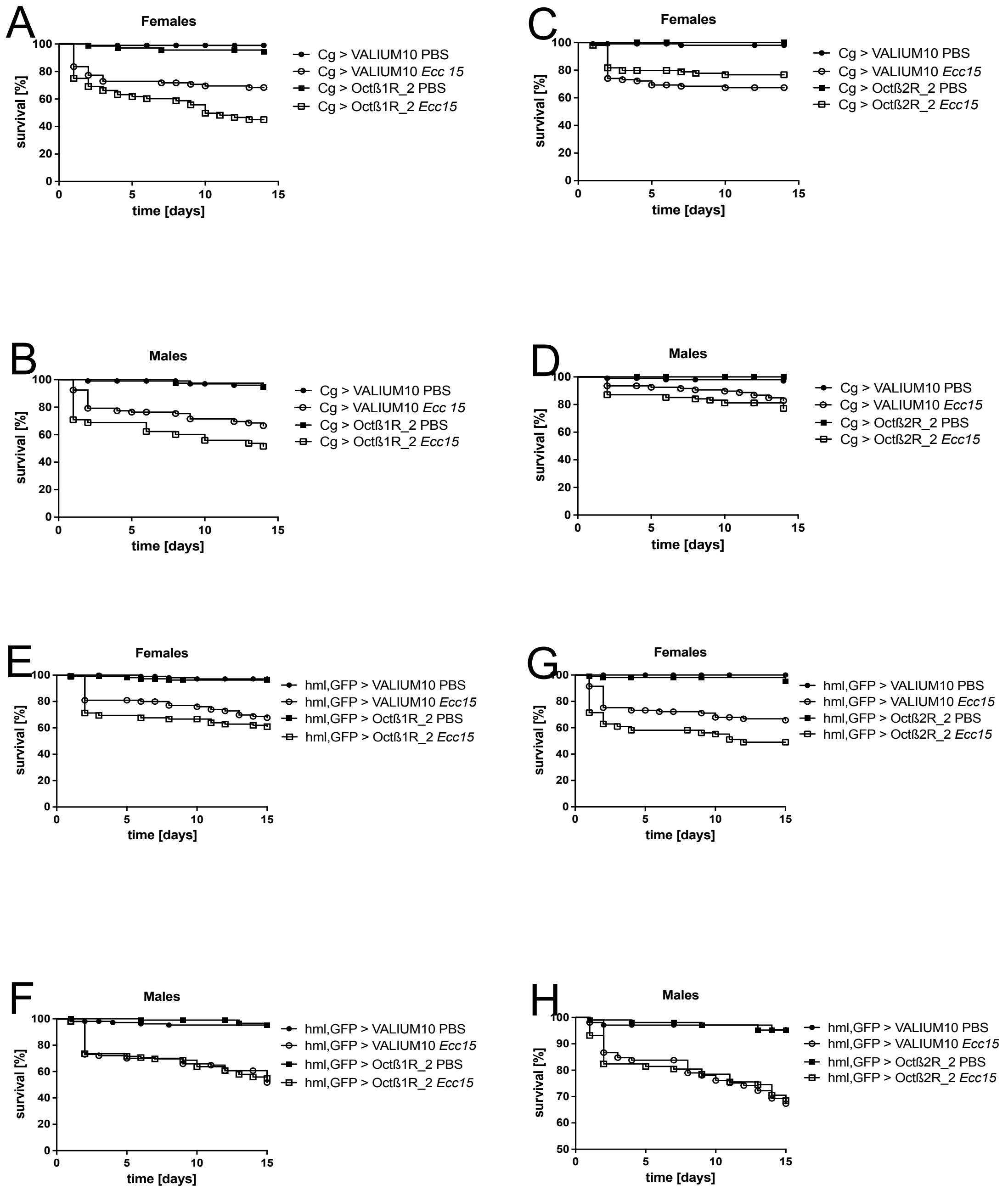

Supplementary Figure 2: Octopamine receptor RNAi and their survival after infection with Ecc15. The survival of *Octβ1R*-RNAi (a-d), *Octβ2R*-RNAi (e-h), and their matching controls (RNAi targeted against an unrelated sequence). RNAi of *Octβ1R* driven in the fat body by Cg-Gal4 for females (a) and males (b). RNAi of *Octβ2R* driven in the fat body by Cg-Gal4 for females (c) and males (d). RNAi of *Octβ1R* driven in the hemocyte by Hml-Gal4 for females (e) and males (f). RNAi of *Octβ2R* driven in the hemocytes by Hml-Gal4 for females (g) and males (h). Each group contained at least 50 flies. Statistics: Log-rank (Mantel-Cox) test.

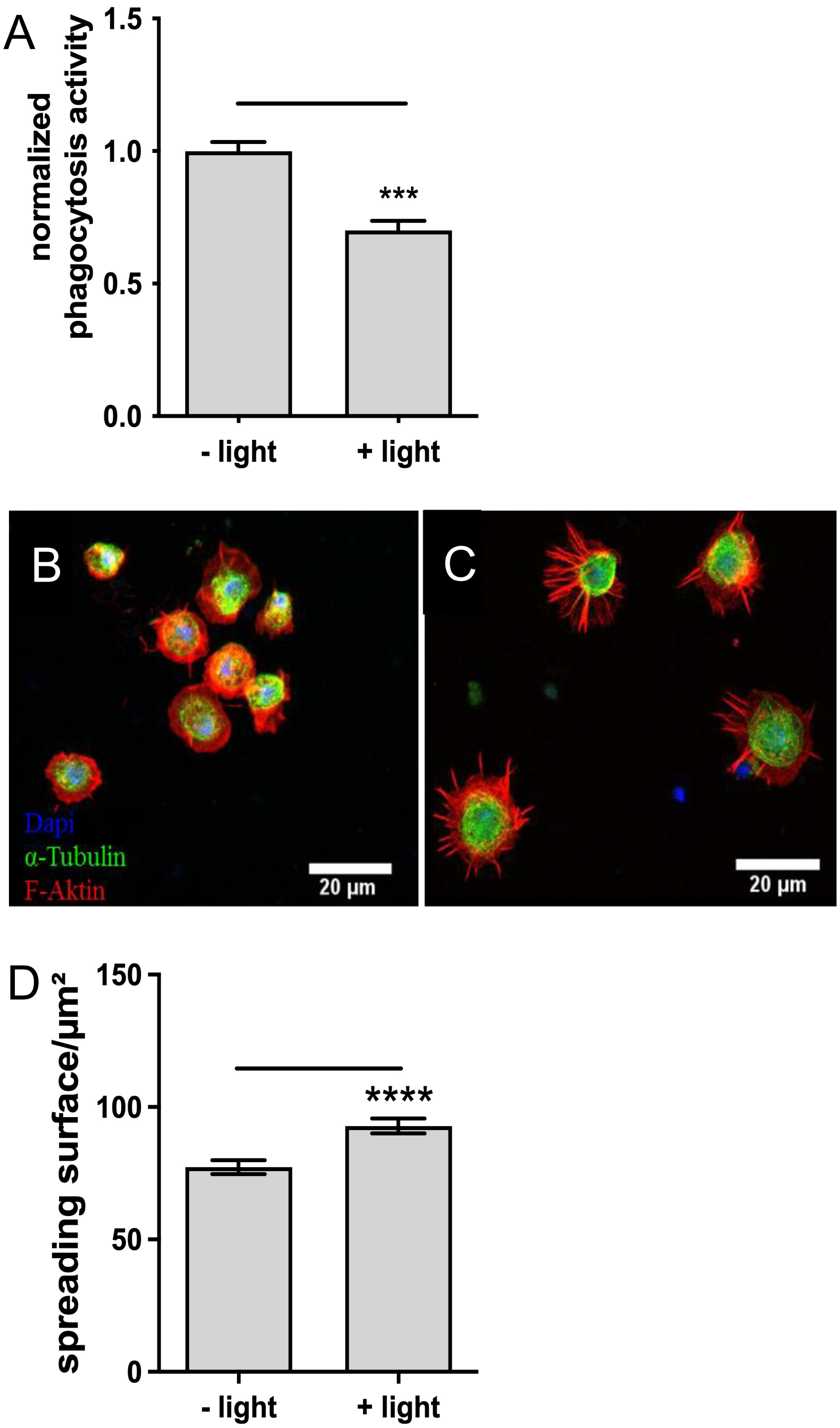

Supplementary Figure 3: Induced increase in cAMP in hemocytes reduced phagocytosis while in increased substrate attachment. Blue-light induced increase in cAMP of hml-Gal4, UAS-bPacII induced reduced phagocytosis of ex vivo hemocytes (a). Substrate spreading response in non-irradiated hemocytes (b) and those that experienced blue light (c). Quantification of the covered areas of non-irradiated and irradiated hemocytes (d). $n \geq 445$, unpaired t-test.

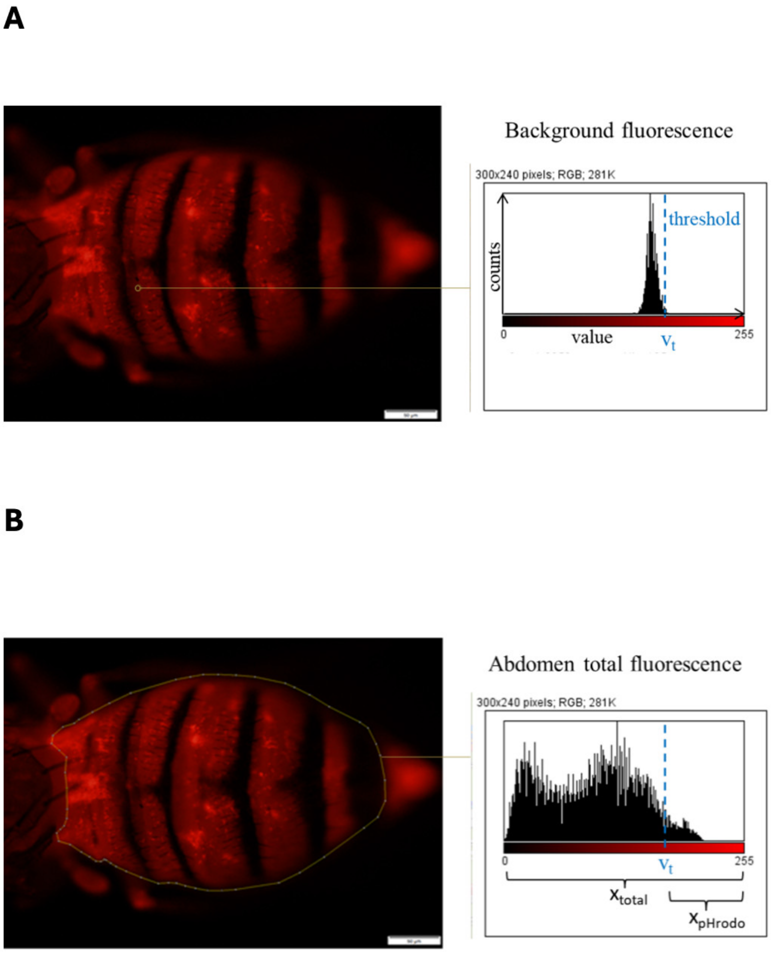

C

$$x_{pHrodo} = \sum_{i=v_t}^{255} v_i \cdot c_i$$

$$x_{total} = \sum_{i=0}^{255} v_i \cdot c_i$$

$$Phagocytic\ index = \frac{x_{pHrodo}}{x_{total}} \cdot 100\%$$

Supplementary Figure 4: Fluorescence images of the adult female abdomen. a: The background fluorescence was determined in a small area in the third abdominal segment (yellow circle). b: The total fluorescence amount was ascertained by encircling the entire abdomen (yellow rim). c: Calculation for the phagocytic index.
